# Molecular Characterization and Phylogenetic Analysis of the 2019 Dengue Outbreak in Wenzhou, China

**DOI:** 10.3389/fcimb.2022.829380

**Published:** 2022-05-19

**Authors:** Axiang Han, Baochang Sun, Zhewei Sun, Xuelian Xu, Qiongying Yang, Danli Xie, Wanchun Guan, Yongliang Lou

**Affiliations:** ^1^ Wenzhou Key Laboratory of Sanitary Microbiology, Key Laboratory of Laboratory Medicine, Ministry of Education, China, School of Laboratory Medicine and Life Sciences, Wenzhou Medical University, Wenzhou, China; ^2^ Department of Clinical Laboratory, Ningbo First Hospital, Ningbo, China; ^3^ Department of Laboratory, Wenzhou Center for Disease Control and Prevention, Wenzhou, China

**Keywords:** dengue virus, whole-genome, phylogenetic analysis, gene mutation, molecular characterization

## Abstract

In 2019, a dengue outbreak occurred with 290 confirmed cases in Wenzhou, a coastal city in southeast China. To identify the origin of the dengue virus (DENV) from this outbreak, viral RNA was extracted from four serum samples and sequenced for whole genome analysis. Then, phylogenetic analysis, gene mutation, secondary structure prediction, selection pressure analysis, and recombination analysis were performed. DENV strains Cam-03 and Cam-11 were isolated from patients traveling from Cambodia, while ZJWZ-18 and ZJWZ-62 strains were isolated from local patients without a record of traveling abroad. The whole genome sequence of all four strains was 10,735 nucleotides long. Phylogenetic tree analysis showed that the four strains belonged to genotype 1 of DENV-1, but the local Wenzhou strains and imported strains clustered in different branches. ZJWZ-18 and ZJWZ-62 were closely related to strain MF033254-Singapore-2016, and Cam-03 and Cam-11 were closely related to strain AB608788-China : Taiwan-1994. A comparison of the coding regions between the local strains and the DENV-1 standard strain (EU848545-Hawaii-1944) showed 82 amino acid mutations between the two strains. A total of 55 amino acid mutations were found between the coding regions of the local and imported strains. The overall secondary structure of the 3′ UTR of the local strains had changed: apparent changes in the head and tail position were observed when compared to DENV-1 standard strain. Furthermore, selection pressure analysis and recombination detection using the 4 isolates and 41 reference strains showed two credible positive selection sites and eight credible recombination events, which warrant further studies. This study may enhance the understanding of viral replication, infection, evolution, virulence, and pathogenicity of DENV.

## Introduction

Dengue is a mosquito-borne disease caused by the dengue virus (DENV) transmitted by *Aedes albopictus* and *A. aegypti* that are mainly distributed in tropical and subtropical climates worldwide, especially in urban and semi-urban areas (Bhatt et al., 2013). DENV infection can lead to varying degrees of clinical symptoms, including mild dengue fever (DF), life-threatening dengue shock syndrome (DSS), and dengue hemorrhagic fever (DHF) (Rodenhuis-Zybert et al., 2010). In the recent decades, the global incidence of dengue has risen rapidly, and approximately half of the world’s population is now at risk, with an estimated 100–400 million infections annually. DENV has become one of the main causes of illness and death in tropical and subtropical regions (WHO, 2015).

DENV, which belongs to the genus *Flavivirus* of the family *Flaviviridae* is an enveloped, positive-sense, single-stranded RNA virus with a genome of 10.6–11.0 kb in length (Chambers et al., 1990; Henchal and Putnak, 1990), consisting of a single open reading frame (ORF) (~ 3400 codons) and 5′ and 3′ untranslated regions (UTRs) (Collins et al., 2018). The ORF can be translated into seven non-structural proteins, namely NS1, NS2a, NS2b, NS3, NS4a, NS4b, and NS5, and three structural proteins, namely C, M, and E proteins (Kuhn et al., 2002). There are four closely related serotypes of DENV (DENV-1, DENV-2, DENV-3, and DENV-4), which share 65–70% sequence homology, but are distinct in antigenicity and genetics (Anoop et al., 2012; Jiang et al., 2018). Each serotype of DENV has further differentiated into numerous genotypes and lineages, owing to the high mutation rate of the viral sequence (Weaver and Vasilakis, 2009). Among the serotypes, DENV-1 is classified into five phylogenetically distinct genotypes (I–V) (Goncalvez et al., 2002). Previous studies have shown that certain genotypes are associated with the proportion of cases with more severe symptoms (Rico-Hesse, 2003; Murray et al., 2013). Therefore, understanding the genotype of the prevalent DENV in different regions is vital for vaccine development and disease surveillance.

During the past few decades, many countries in Asia, including India, Vietnam, Myanmar, Japan, Indonesia, Thailand, Malaysia, Cambodia, Pakistan, and Laos, have frequently reported epidemic dengue outbreaks (Quam et al., 2016; Kyaw et al., 2017; Gintarong et al., 2018; Phadungsombat et al., 2018; Kar et al., 2019; Sasmono et al., 2019). Due to globalization, many provinces in mainland China, including Guangdong, Guangxi, Yunnan, Fujian, Henan, and Zhejiang, have seen an increase in DENV infection cases in recent years, (Chen and Liu, 2015; Sun et al., 2017; Yue et al., 2019). Wenzhou city, located in the southeastern part of Zhejiang Province, has several commercial trades, labor exports, tourism, and cultural exchanges with foreign countries every year. In 2014, a large-scale dengue outbreak occurred in southern China, while five cases were reported in Wenzhou (Wang et al., 2015). According to the statistics reported by the Wenzhou Center for Disease Control and Prevention (CDC), sporadic imported cases of DENV infection have been documented annually in Wenzhou city in recent years.

In 2019, a sudden large dengue epidemic with more than 290 reported cases occurred in Wenzhou. We analyzed the strain sequence of DENV from this year to trace the origin and understand the molecular characteristics of the virus. In this study, we first confirmed the origin of this epidemic in Wenzhou and compared the whole genome sequence of DENV-1 isolated from the 2019 outbreak.

## Materials and Methods

### Ethics Statement

The study protocol was approved by the Institutional Ethics Committee of the Wenzhou CDC. Written informed consent was obtained from all participants.

### Sample and Virus RNA Extraction

**Figure 1:**
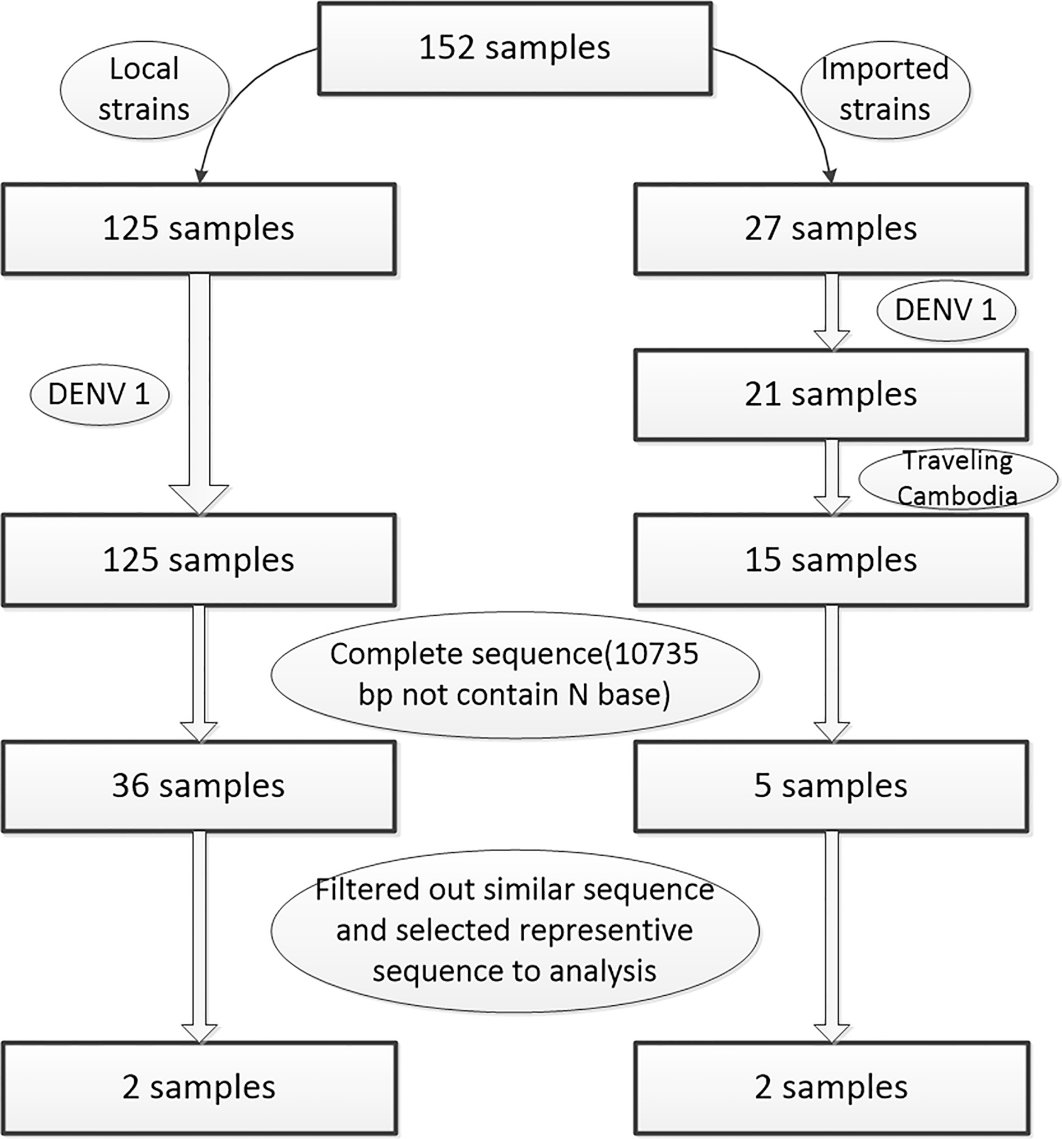
Flowchart of sample filtering process.

One hundred fifty two serum samples were collected by the Wenzhou CDC from various hospitals in Wenzhou during 2019. All 152 patients were dengue fever, no patients were dengue hemorrhagic fever and dengue shock syndrome. Viral RNA was extracted using the MagNA Pure LC Total Nucleic Acid Isolation Kit (Roche, Germany; REF 03038505001) according to the manufacturer’s instructions. The RNA products were sequenced by Magigene Company (Guangzhou, China) (Wang et al., 2019). By PCR and BLAST analysis, serotype 1 strains were the mainly in 2019, the serotype distributions of DENV strains in 2019 were shown in **Supplementary Table 1**. Amongst, 15 samples traveling from Cambodia are mainly of 27 samples traveling abroad strains. The other samples were filtered out, such as the sequences with high similarity, uncompleted, lacking patients information, and other serotype (e.g., serotype2 and serotype3). Then sequences from two patients without a record of traveling abroad and two patients traveling from Cambodia were selected for further whole genome and molecular characterization analysis of DENV (**Figure 1**).

### Sequencing Library Preparation and Genome Sequencing

RNA concentration and purity were evaluated using Nanodrop One (NanoDrop Technologies, Wilmington, DE, USA), and RNA integrity was determined using agarose gel electrophoresis. The REPLI-g Cell WGA & WTA Kit (Qiagen, Germany) was used for whole transcriptome amplification to obtain double-stranded cDNA for samples with low concentrations, following the manufacturer’s instructions. Library preparation was performed using the ALFA-SEQ DNA Library Prep Kit kit following the manufacturer’s instructions and sequenced on an Illumina NovaSeq-PE150.

The raw sequencing data were first filtered using Soapnuke (https://github.com/BGI-flexlab/SOAPnuke) to remove low-quality reads and obtain clean data. Then, clean reads were aligned to a human genome reference using the BWA software (v0.7.17) and filtered to remove host contamination that could affect the subsequent data analysis. The clean reads without host contamination were aligned to different genotypes of dengue sequences obtained from the NCBI database, and the sequence with the highest similarity was determined as the reference genome. Clean reads were aligned to the reference sequence using BWA (v0.7.17) and SamTools (v0.7.17) software to obtain a consensus sequence.

### Genomic Characterization and Phylogenetic Analysis

Multiple alignments of nucleotide (nt) sequences and translated amino acid sequences were performed using MAFFT v7.407 (Katoh and Standley, 2013). The UTR secondary structure was predicted using the RNA fold web server by default. (http://rna.tbi.univie.ac.at/cgi-bin/RNAWebSuite/RNAfold.cgi). Phylogenetic analysis, based on the DENV-1 whole genome sequence, was performed using the maximum likelihood method (Bootstrap1000, Tamura-Nei model) in MEGA-X software version 10.1.8 (Kumar et al., 2018). The reference DENV-1 whole genome sequences used to construct the phylogenetic tree were obtained from the GenBank sequence database from the following countries under the corresponding accession numbers (**Table S2**): China (JQ048541, MF681693, KP772252, KP723473, KU365900, KU094071, DQ193572, AB608788, and KT827374), Singapore (EU081262, MF033254, GQ357692, and JN544411), Thailand (HG316481, FJ850068, and AF180817), Japan (AB178040, AB074760, and AB204803), Laos (KC172829, KC172834, and KC172835), Hawaii (EU848545, DQ672562, and DQ672564), Vietnam (JQ045626 and FJ390386), Cambodia (FJ639677 and AF309641), India (KJ755855 and KF289072), Myanmar (AY726553 and AY726554), Malaysia (JN697058), Sri Lanka (HQ891316), Indonesia (KC762646), South Korea (KP406802), Chile (EU863650), South Pacific (JQ915072), Western Pacific (U88535), and the USA (FJ390379). We used AF180817-Thailand-1964 as outgroup.

### Selection Pressure Analysis

The online prediction web server Datamonkey (http://www.datamonkey.org/) was used to assess the influence of the selection pressure acting on every codon in the coding sequence of the viruses. After preparing a dataset consisting of 45 complete coding sequences of DENV-1 (41 reference sequences and 4 obtained sequences from the sera samples (**Supplementary Table 2**), four different approaches, namely fixed effects likelihood (FEL) (Kosakovsky Pond and Frost, 2005), mixed effect model of evolution (MEME) (Murrell et al., 2012), single-likelihood ancestor counting (SLAC) (Kosakovsky Pond and Frost, 2005), and Fast, Unconstraited Bayesian AppRoximation (FUBAR) (Murrell et al., 2013), were executed with default parameters to estimate the non-synonymous to synonymous substitution rate ratio (ω) per codon site. Significant values were assessed using the following criteria: posterior probability > 0.9 in FUBAR, and *p* value < 0.1 in FEL, MEME, and SLAC.

### Recombination Analysis

The prepared dataset (**Supplementary Table 2**) of 45 complete coding sequences of DENV-1 was imported to the Recombination Detection Program 4 (version 4.101) (Martin et al., 2015) and subjected for recombination analysis using seven methods, including RDP (Martin and Rybicki, 2000), GENCONV (Padidam et al., 1999), BootScan (Martin et al., 2005), MaxChi (Smith, 1992), Chimaera (Posada and Crandall, 2001), SiScan (Gibbs et al., 2000), and 3Seq (Lam et al., 2018). The recombinant event was determined using the following criteria: recombinant signal was detected using at least two methods and *p* value < 0.05. The resulting p-value was adjusted by applying the Benjamini and Hochberg’s approach to limit the false discovery rate (FDR).

## Results

### Nucleotide Sequence Analysis of the Full-length Sequences of All Four Isolates

The clean reads of all four isolates were aligned to the reference sequence of DENV-1 (NC_001477.1). All isolates have a full-length sequence of 10,735 nucleotides; and the 5′ UTR and 3′ UTR were 94 and 465 nucleotides long, respectively. Furthermore, the ORF was 10,176 nucleotides long, encoding for 3,392 amino acids, and located between nucleotides 95 and 10,270. The nucleotide composition of the coding region of the whole viral genome sequence is shown in **Table 1**. Notably, the two local epidemic viruses in Wenzhou were highly similar in sequence.

**Table 1:** Statistical analysis of nucleotides in the coding region of the whole genome sequence.

	A (%)	G (%)	C (%)	T (%)	Length	C+Gcontent
Cam-03	3252 (31.96%)	2624 (25.79%)	2105 (20.68%)	2195 (21.57%)	10176	46.47%
Cam-11	3251 (31.95%)	2619 (25.74%)	2115 (20.78%)	2191 (21.53%)	10176	46.52%
ZJWZ-18	3264 (32.08%)	2621 (25.75%)	2094 (20.58%)	2197 (21.59%)	10176	46.33%
ZJWZ-62	3265 (32.09%)	2620 (25.74%)	2096 (20.60%)	2195 (21.57%)	10176	46.34%
EU848545	3226 (31.70%)	2652 (26.06%)	2094 (20.58%)	2204 (21.66%)	10176	46.64%

### Phylogenetic Analysis

**Figure 2:**
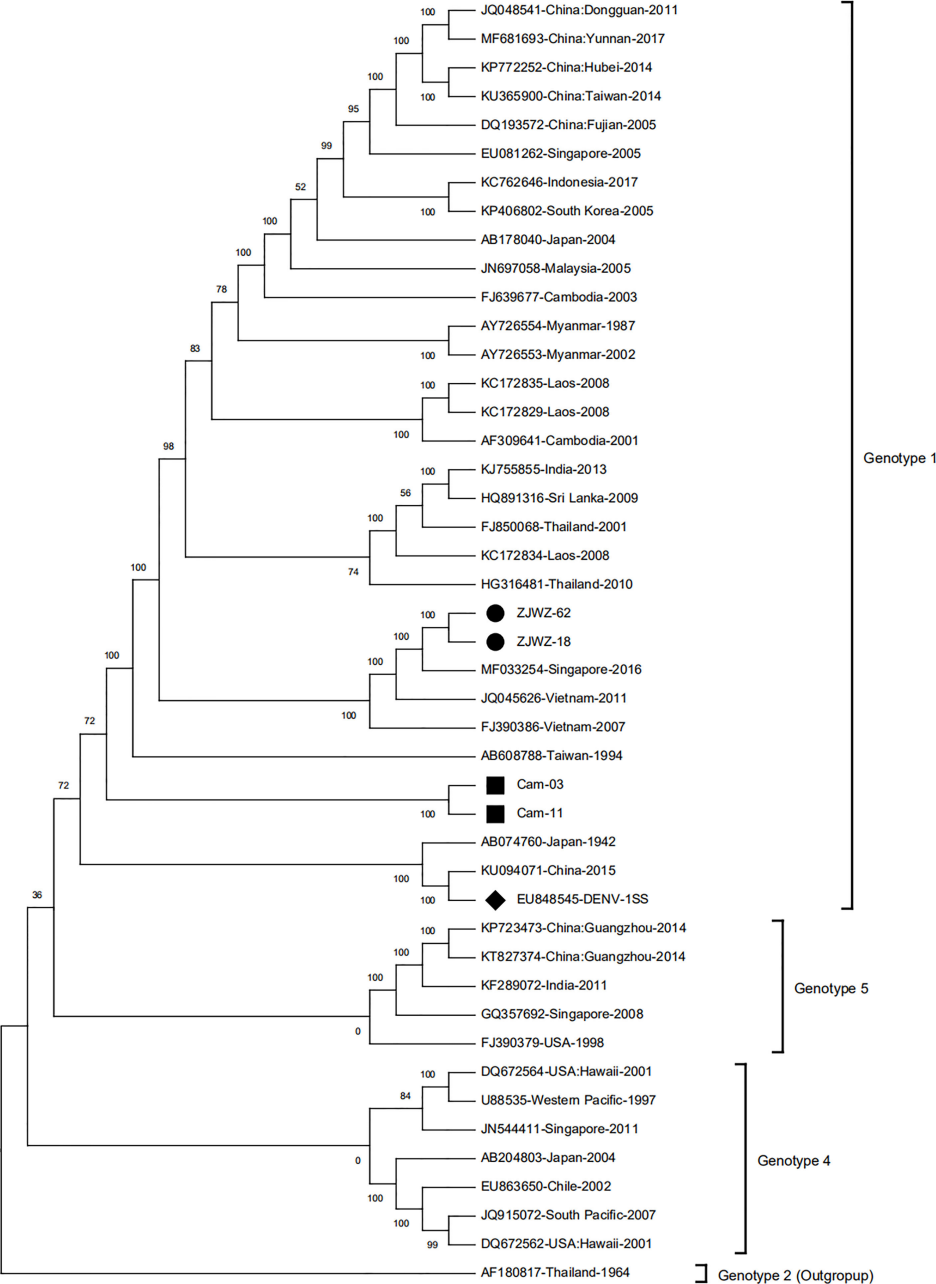
Phylogenetic analysis based on whole genome sequence. ● represent Wenzhou local strains, ◼ represent strain with traveling Cambodia, ♦ represent standard strain (Hawaii-1944).

The whole genome sequences of strains isolated from patients traveling from Cambodia (Cam-03 and Cam-11) and those isolated locally (ZJWZ-18 and ZJWZ-62) were aligned with other 41 representative DENV-1 sequences obtained from GenBank. As observed in the phylogenetic tree (**Figure 2**), the four isolates belonged to genotype 1 of DENV-1. Cam-03 and Cam-11 were clustered individually, and ZJWZ-18 and ZJWZ-62 were closely clustered with DENV-1 MF033254-Singapore-2016, JQ045626-Vietnam-2011, and FJ390386-Vietnam-2007. Therefore, the two local isolates may be derived from the Singapore strain (MF033254) or a Vietnam strain (JQ045626 or FJ390386).

### Base Substitution and Amino Acid Mutation Analysis of Cam-03, Cam-11, ZJWZ-18, and ZJWZ-62 Coding Sequences

The coding sequences of the four strains were compared with the DENV-1 standard strain (EU848545-HAWAII-1944), as shown in **Table 2**. The number of base mutations in Cam-03, Cam-11, ZJWZ-18, and ZJWZ-62 were 540, 557, 597, and 598, respectively. The highest mutant region from ZJWZ-18 and ZJWZ-62 was the prM protein, and that from Cam-03 and Cam-11 was the NS4A protein. The difference in the overall mutation rates of the four strains was not large.

**Table 2:** Nucleotide and amino acid substitutions in the translated regions.

		C	prM/M	E	NS1	NS2A	NS2B	NS3	NS4A	NS4B	NS5	Total
Cam-03 vs. EU848545	Base substitution	13	36	85	66	40	24	92	34	32	118	540
	Base substitution rate	3.83	7.32	5.70	6.25	6.12	6.15	4.95	7.56	4.28	4.38	5.31
	AA substitutions	5	7	14	10	10	2	9	3	3	15	78
	AA substitution rate	4.42	4.27	2.82	2.84	4.59	1.54	1.45	2.00	1.20	1.67	2.30
Cam-11 vs. EU848545	Base substitution	11	37	88	67	40	24	95	34	35	126	557
	Base substitution rate	3.24	7.52	5.90	6.34	6.12	6.15	5.12	7.56	4.69	4.67	5.47
	AA substitutions	3	8	14	10	10	2	9	3	4	12	75
	AA substitution rate	2.65	4.88	2.82	2.84	4.59	1.54	1.45	2.00	1.61	1.33	2.21
ZJWZ-18 vs. EU848545	Base substitution	16	39	94	79	43	23	97	21	38	147	597
	Base substitution rate	4.72	7.93	6.3	7.48	6.57	5.9	5.22	4.67	5.09	5.45	5.87
	AA substitutions	6	8	17	10	6	2	12	2	3	16	82
	AA substitution rate	5.31	4.88	3.42	2.84	2.75	1.54	1.94	1.33	1.2	1.78	2.42
ZJWZ-62 vs. EU848545	Base substitution	16	39	93	79	43	23	99	21	38	147	598
	Base substitution rate	4.72	7.93	6.24	7.48	6.57	5.9	5.33	4.67	5.09	5.45	5.88
	AA substitutions	6	8	16	10	6	2	11	2	3	16	80
	AA substitution rate	5.31	4.88	3.22	2.84	2.75	1.54	1.78	1.33	1.2	1.78	2.36

**Figure 3:**
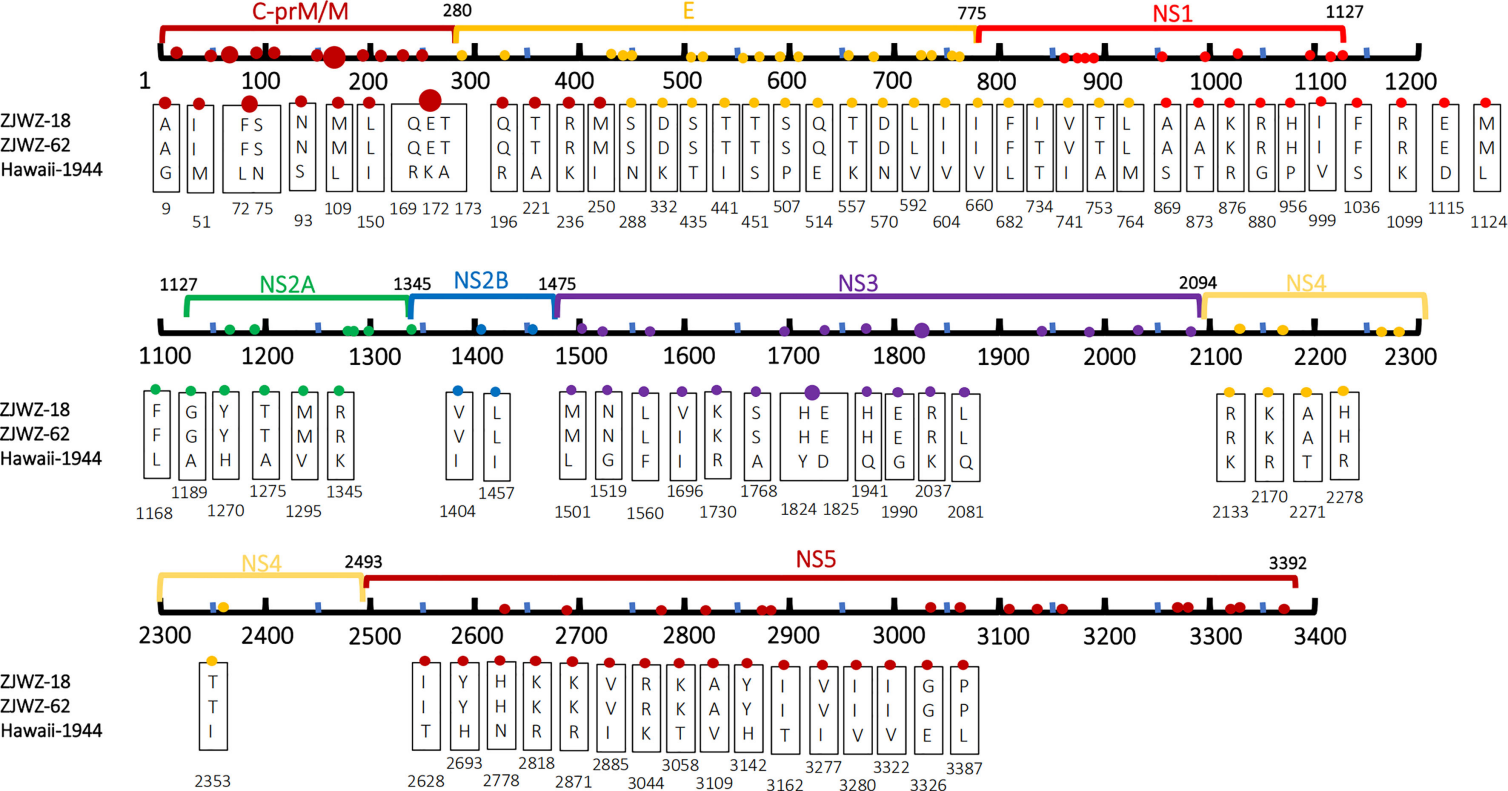
Amino acid mutations between CDS of Wenzhou local strains and Hawaii strains (1944). The labeled numbers represent the amino acid position.

As shown in **Figure 3**, compared with the standard strains, the two local Wenzhou strains had a total of 82 amino acid mutations. ZJWZ-18 and ZJWZ-62 had 31 and 30 amino acid mutations, respectively, in the structural protein regions. Meanwhile, ZJWZ-18 and ZJWZ-62 had 51 and 50 amino acid mutations, respectively, in the non-structural protein regions. ZJWZ-18 had two more amino acid site mutations than ZJWZ-62: T734I located in the E protein region and I1696V located in the NS3 protein region. The mutation rate of amino acids in structural proteins was higher than that in non-structural proteins.

**Figure 4:**
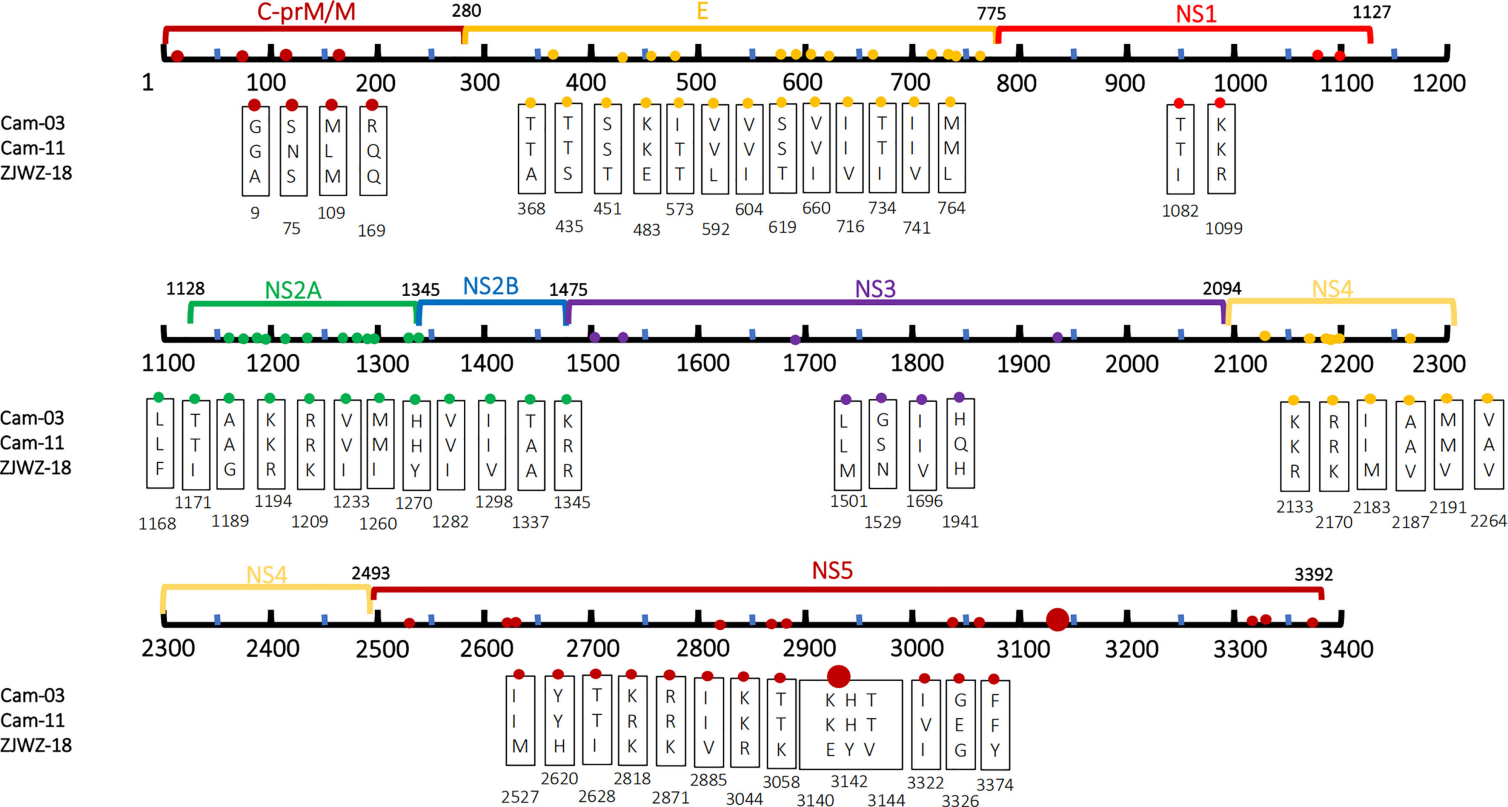
Amino acid mutations between CDS of Wenzhou local strain and 2 strains with traveling Cambodia. The labeled numbers represent the amino acid position.

Compared with the Wenzhou local strains, Cam-03 and Cam-11 had a total of 55 amino acid mutations (**Figure 4**). Moreover, 13 amino acid mutations between the two imported strains, including five mutations in the structural protein region: N75S, L109M, Q169R, T573I, and L592V; and eight mutations in the non-structural protein regions: A1337T, R1345K, S1519G, Q1941H, A2264V, R2818K, V3322I, and E3326G, were observed.

**Figure 5:**
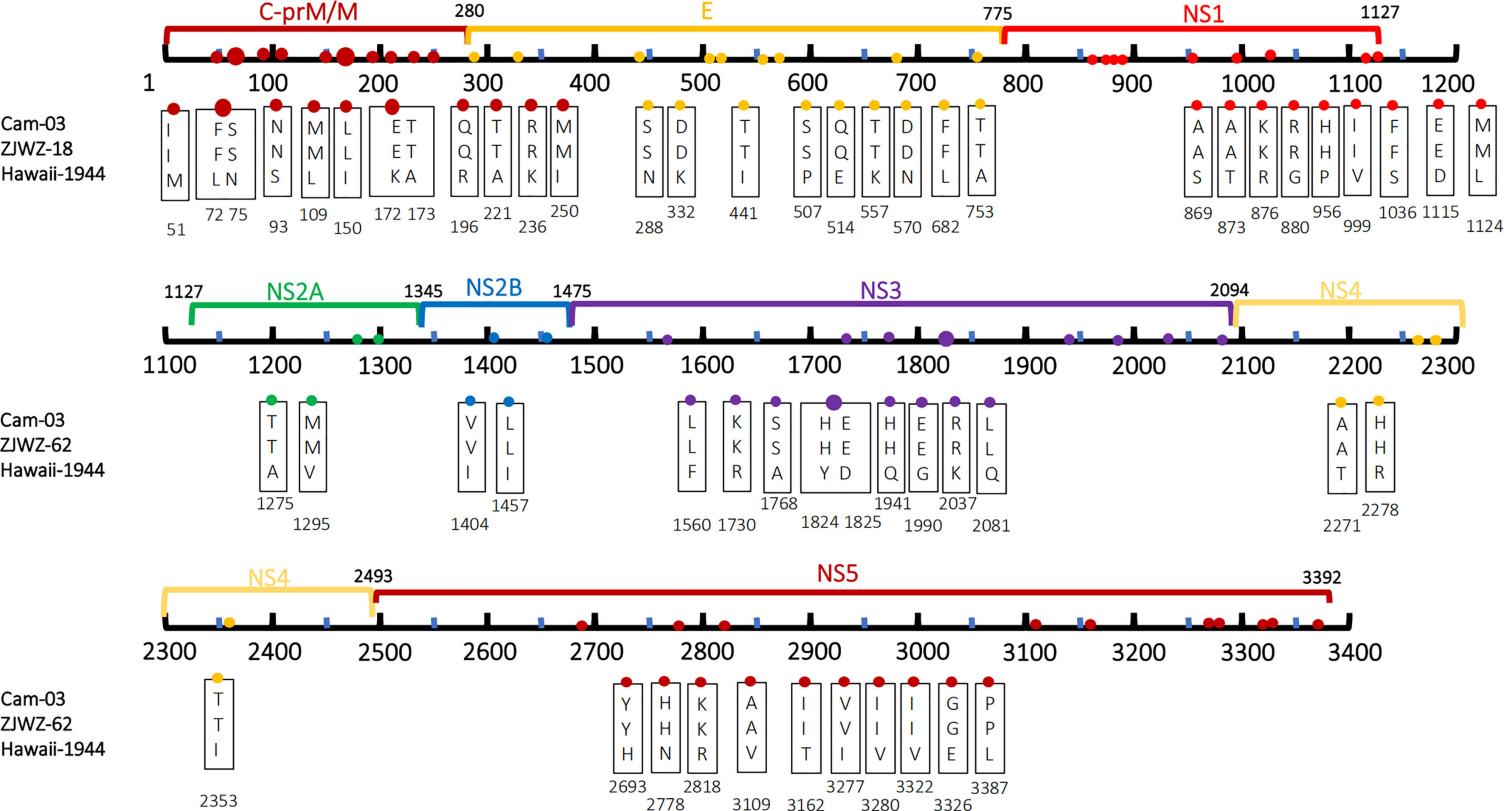
Amino acid mutations both in Wenzhou local strain and traveling Cambodia strain compared with standard strain. The labeled numbers represent the amino acid position.

As the **Figure 5**, we have found 56 Non-synonymous amino acid mutations in both local and Cambodia strains compared to the standard strains. In the E protein, nine amino acid mutations were observed: N288S, K332D, I441T, P507S, E514Q, K557T, N570D, L682F, A753T.

**Figure 6:**
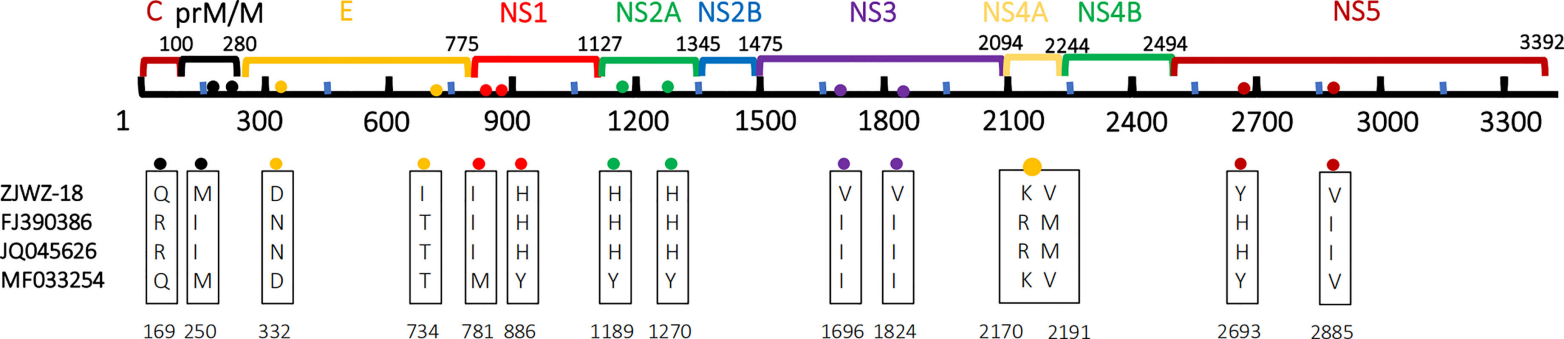
Amino acid mutations between CDS of Wenzhou local strain and 3 closed phylogenetic strains. The labeled numbers represent the amino acid position.

Phylogenetic analysis revealed that a total of 14 amino acid mutations occurred in the coding sequence of the local isolates (**Figure 6**), as compared to the closely-related Singapore strain (MF033254-Singapore-2016) and Vietnam strains (FJ390386-Vietnam-2007, JQ045626-Vietnam-2011). Specifically, compared to the 2016 Singapore strains, seven amino acid mutations were observed: one in the structural protein coding region,T734I (E), and six in the non-structural protein coding region, namely M781I (NS1), Y886H (NS1), Y1189H (NS2A), Y1270H (NS2A), I1696V (NS3), and I1824V (NS3). Compared with the 2007 Vietnam strains, 10 amino acid mutations were observed: four were distributed in the structural protein coding region, namely R169Q(prM/M), I250M(prM/M), N332D(E), and T734I(E), and six were distributed in the non-structural protein coding region, namely I1696V(NS3), I1824V(NS3), R2170K(NS4A), M2191V (NS4A), H2693Y (NS5), and I2885V (NS5). Similarly, these results were observed when the local strains are compared to the 2011 Vietnam strain.

### Prediction of the RNA Secondary Structure Based on the Untranslated Regions

**Figure 7:**
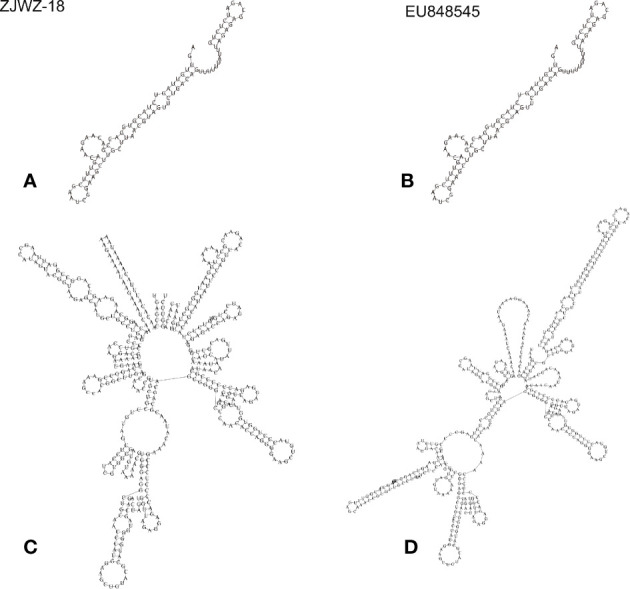
The predicted secondary structure of UTRs of the Wenzhou local strain and standard strain. **(A, C)** represent Wenzhou local strain’s 5′ UTR and 3′ UTR respectively. **(B, D)**represent the standard strain’s 5 UTR and 3′ UTR, respectively.

The Wenzhou local strain lacked a single nucleotide (T) at position 79 compared to the standard strain, and there were no base substitutions, insertions, or deletions in the remaining positions. Therefore, the change in the predicted RNA secondary structure of the 5′ UTR was only marginal (**Figures 7A, B**). As compared to the 3′ UTR of the standard strain, the Wenzhou local strain had 12 substitutions (nt 10276[T→C], nt 10286[C→T], nt 10293[C→T], nt 10297[G→A], nt 10382[T→C], nt 10408[G→A], nt 10418[T→C], nt 10467[C→T], nt 10536[A→G], nt 10542[C→T], nt 10621[C→T], nt 10702[G→A]) and 2 inserts (nt 10455[G], nt 10479[A]). RNA secondary structure prediction showed that some nucleotide substitutions in the 3′ UTR of the local strain led to a change in the secondary structure. The predicted structure had a long stem in the initial, a shorter stem loop in the terminal, and fewer loops than the standard strain, which had a loop in the initial, a longer stem loop in the terminal, and more loops **Figures 7C, D**.

### Selection Pressure Analysis

Most codons in the coding sequence of DENV-1 were found to be under negative selection. A total of four sites were detected to be under positive selection pressure by more than two out of the four methods (**Table 3**). Moreover, sites 869 and 3144 of NS1 and NS5, respectively, were under positive selection by at least three different methods.

**Table 3:** Selection pressure analysis of the ORF (3391 codons) of DENV-1 using FEL, MEME, SLAC, and FUBAR.

Amino acid site and protein region	FEL		MEME		SLAC		FUBAR	
	ω	*P* value	ω	*P* value	ω	*P* value	ω	Posterior probability
E protein								
332	Infinity	0.076	Infinity	0.100	Infinity	0.331	2.550	0.752
NS1 protein								
869	Infinity	0.011	Infinity	0	Infinity	0.105	9.726	0.957
NS3 protein								
1825	Infinity	0.063	Infinity	0.080	Infinity	0.229	4.171	0.820
NS5 protein								
3144	5.330	0.065	8.843137	0.080	3.972	0.145	6.104	0.911

### Recombination Analysis

The P-value and Padj-value were listed in **Supplementary Tables 3**, **4**. Recombination analysis revealed 10 potential recombination events (**Table 4**). Among the detected events, four recombination events of Cam-03 and three recombination events of Cam-11 conformed to at least six methods, so these seven potential recombination events were considered to be highly credible.

**Table 4:** Recombination analysis of DENV-1 by RDP package.

EVENTNo.	Recombinant sequence	Break point position	Parental sequence	Detection methods						
		Begin/End	Major/Minor	RDP	GENECONV	BootScan	Maxchi	Chimaera	SiScan	3Seq
1	Cam-11	3994/5583	U88535/ZJWZ-62	+	+	+	+	+	+	+
2	Cam-11	408/1070	U88535/ZJWZ-18	+	+	+	+	+	+	+
3	Cam-11	2549/3165	U88535/ZJWZ-18	+	+	+	+	+	+	+
4	Cam-03	9540/10066	U88535/ZJWZ-18	+	+	+	+	+	+	–
5	Cam-03	5670/6106	Cam-11/ZJWZ-18	+	+	+	+	+	+	+
6	Cam-03	8001/8536	U88535/ZJWZ-18	+	+	+	+	+	+	+
7	Cam-03	202/407	Cam-11/JQ045626	+	+	–	+	+	–	+
8	Cam-03	6756/7096	U88535/ZJWZ-18	+	+	+	+	+	–	+
9	Cam-11	1550/1804	U88535/ZJWZ-18	+	–	+	–	–	–	+
10	KJ755855	7664/7924	DQ193572/Unknow	+	–	–	+	–	+	–

## Discussion

Wenzhou, located in the south of Zhejiang Province, China, have frequent exchanges in trade, culture, and tourism with Southeast Asia, Europe, and other countries around the world. Wenzhou’s climate and geographical environment are suitable for mosquito breeding. Therefore, it provides favorable conditions for the spread of DF. Interestingly, there have been sporadic cases of imported DF every year (Ren et al., 2018). However, very few studies have focused on the molecular information of the reported dengue virus in Wenzhou. In this study, the whole genome sequence, genotype, and molecular characterization of the Wenzhou local strain were analyzed in this study, which are essential for vaccine development, disease surveillance, and epidemiology.

Phylogenetic analysis, constructed using the whole viral genome sequences, demonstrated that the four isolates, two imported and two local strains, belonged to genotype 1 of DENV-1, and the local strains were closely clustered with DENV-1 MF033254-Singapore-2016, JQ045626-Vietnam-2011, and FJ390386-Vietnam-2007, suggesting that they likely originated from Southeast Asian countries. This result was also consistent with the information from the Wenzhou Exit-Entry Administration, which is mainly about personnel exchanges between Southeast Asian countries and Wenzhou city.

The virulence of flavivirus strains is affected by multiple amino acid sites. Several studies have shown that some mutations of special amino acid sites have a significant impact on the virulence, replication, and infectivity of flaviviruses. In the PrM protein of Zika virus, a single mutation (S139N) enhanced the infectivity of the virus and increased fetal microcephaly incidence and mortality rate of newborn mice (Yuan et al., 2017). The mutation of T155I in the E protein region of DENV-1 and DENV-4 ablated a conserved glycosylation site in E protein and promoted the virulence of the virus (Kawano et al., 1993; Puri et al., 1997). A single mutation (G66A) in the NS2A protein of the Japanese encephalitis-live vaccine virus attenuated the formation of NS1 and reduced neurovirulence in mice (Ye et al., 2012). In this study, compared with the standard strain, the mutation rate of structural proteins of the two local strains was higher than that of the non-structural protein, which is consistent with the previous research results of Jiang et al. (2018). As previously described, the mutation of T155I in the E protein region of DENV-1 and DENV-4 promoted the virulence of the virus (Kawano et al., 1993; Puri et al., 1997). Thus, in our study, this mutation may enhance the virulence of the virus. The effects of other amino acid substitutions on replication, translation, infection, virulence, and other functions of the local virus strain require further investigation.

The UTRs at both ends of the viral genome play an important role in replication, translation, and synthesis of RNA (Chiu et al., 2005; Leardkamolkarn et al., 2010). The 3′ UTR is divided into three regions based on conservative regions: (1) a variable area; (2) a cyclization sequence motif and stable stem-loop structure; and (3) a region between the variable and 3-terminal regions (Bryant et al., 2005). As previously reported, the short variable region in the 3′ UTR of the Japanese encephalitis virus did not affect its growth *in vitro* or its pathogenicity in mice (Kato et al., 2012). However, another report showed that the variable region in the 3′ UTR of DENV can promote viral replication in baby hamster kidney cells (Alvarez et al., 2005). In our study, some nucleotide substitutions were observed in the 3′ UTR compared to the standard strains. Moreover, the RNA secondary structure was altered. The effects of these substitutions on DENV replication, translation, and synthesis require further study.

The selection pressure on the virus can reflected whether the mutations of the virus can be preserved, ω > 1 indicated that the site is under a positive selection pressure; ω ≤ 1 indicated that the site is subject to neutral and purification selection. Selection pressure analysis were indispensable for vaccine development. The selection pressure analysis showed that most codons were under negative selection, which was attributed to the evolutionary constraints of arboviruses (Holmes and Twiddy, 2003). However, four positive selection sites were screened, of which two sites were selected based on important evidence. The effects of these positively selected sites require further investigation.

Gene recombination is a molecular mechanism that plays an important role in the evolution, transmission, and genetic diversity of DENV (Worobey et al., 1999; Simon-Loriere and Holmes, 2011). In our study, 10 recombination events were identified that were mainly concentrated between two imported strains from patients traveling from Cambodia and two Wenzhou local strains. We speculated the human hosts or mosquito hosts of the four isolates were co-infected. Gene recombination increases the difficulty of vaccine research; thus, it is worthy of attention of vaccine researchers.

This study is the first report on the whole genome sequences of DENV from Wenzhou, China in 2019. These results provide a reference for further investigation of the infection, proliferation, virulence, epidemiology, and vaccine development of DENV.

## Data Availability Statement

The datasets presented in this study can be found in online repositories. The names of the repository/repositories and accession number(s) can be found in the article/**Supplementary Material**.

## Ethics Statement

The studies involving human participants were reviewed and approved by Institutional Ethics Committee of the Wenzhou Centers for Disease Control and Prevention. The patients/participants provided their written informed consent to participate in this study.

## Author Contributions

Conceived and designed the experiments: AH, WG, BS, and YL. Performed the experiments: AH, XX, and QY. Analyzed the data: AH, SZ, WG, BS, and DX. Wrote and revised the paper: AH, WG, XX, BS, DX, and YL. All authors contributed to the article and approved the submitted version.

## Funding

This research was supported by the National Major Infectious Disease Prevention Projects(2018ZX10201001).

## Conflict of Interest

The authors declare that the research was conducted in the absence of any commercial or financial relationships that could be construed as a potential conflict of interest.

## Publisher’s Note

All claims expressed in this article are solely those of the authors and do not necessarily represent those of their affiliated organizations, or those of the publisher, the editors and the reviewers. Any product that may be evaluated in this article, or claim that may be made by its manufacturer, is not guaranteed or endorsed by the publisher.

## Supplementary Material

Supplementary Table 1 The serotype distributions of DENV strains in 2019 by PCR.

Supplementary Table 2 Information of reference sequences.

Supplementary Table 3 P-value of recombination analysis.

Supplementary Table 4 Padj-value of recombination analysis.
