# Supplementary material for: Molecular Characterization and Phylogenetic Analysis of the 2019 Dengue Outbreak in Wenzhou, China
Source: Front Cell Infect Microbiol. 2022 May 19;12:829380. doi: 10.3389/fcimb.2022.829380 (PMC9161089; doi:10.3389/fcimb.2022.829380)
Supplement: Supplementary Table 1 — The serotype distributions of DENV strains in 2019 by PCR. [file Table_1.docx]

**TABLE S1.** The serotype distributions of DENV strains in 2019 by PCR.

|  | DENV-1 | DENV-2 | DENV-3 | DENV-4 | Total |
| --- | --- | --- | --- | --- | --- |
| Numbers | 143 | 6 | 3 | 0 | 152 |
