# Supplementary material for: Molecular Characterization and Phylogenetic Analysis of the 2019 Dengue Outbreak in Wenzhou, China
Source: Front Cell Infect Microbiol. 2022 May 19;12:829380. doi: 10.3389/fcimb.2022.829380 (PMC9161089; doi:10.3389/fcimb.2022.829380)
Supplement: Supplementary Table 2 — Information of reference sequences. [file Table_2.docx]

**TABLE S2.** Information of reference sequences .

|  | Accession No. | Country-Year | Serotype | Genotype |
| --- | --- | --- | --- | --- |
| 1 | JQ048541 | China:Dongguan-2011 | DENV-1 | Genotype-1 |
| 2 | KP772252 | China:Hubei-2014 | DENV-1 | Genotype-1 |
| 3 | KU365900 | China:Taiwan-2014 | DENV-1 | Genotype-1 |
| 4 | DQ193572 | China:Fujian-2005 | DENV-1 | Genotype-1 |
| 5 | EU081262 | Singapore-2005 | DENV-1 | Genotype-1 |
| 6 | MF681693 | China:Yunnan-2017 | DENV-1 | Genotype-1 |
| 7 | JN697058 | Malaysia-2005 | DENV-1 | Genotype-1 |
| 8 | FJ639677 | Cambodia-2003 | DENV-1 | Genotype-1 |
| 9 | AF309641 | Cambodia-2001 | DENV-1 | Genotype-1 |
| 10 | FJ850068 | Thailand-2001 | DENV-1 | Genotype-1 |
| 11 | HQ891316 | Sri Lanka-2009 | DENV-1 | Genotype-1 |
| 12 | KJ755855 | India-2013 | DENV-1 | Genotype-1 |
| 13 | HG316481 | Thailand-2010 | DENV-1 | Genotype-1 |
| 14 | AB074760 | Japan-1942 | DENV-1 | Genotype-1 |
| 15 | KC762646 | Indonesia-2017 | DENV-1 | Genotype-1 |
| 16 | KP406802 | South Korea-2005 | DENV-1 | Genotype-1 |
| 17 | AB178040 | Japan-2004 | DENV-1 | Genotype-1 |
| 18 | AY726554 | Myanmar-1987 | DENV-1 | Genotype-1 |
| 19 | AY726553 | Myanmar-2002 | DENV-1 | Genotype-1 |
| 20 | KC172829 | Laos-2008 | DENV-1 | Genotype-1 |
| 21 | KC172835 | Laos-2008 | DENV-1 | Genotype-1 |
| 22 | KC172834 | Laos-2008 | DENV-1 | Genotype-1 |
| 23 | AB608788 | China:Taiwan-1994 | DENV-1 | Genotype-1 |
| 24 | EU848545 | USA:Hawaii-1944 | DENV-1 | Genotype-1 |
| 25 | KU094071 | China-2015 | DENV-1 | Genotype-1 |
| 26 | MF033254 | Singapore-2016 | DENV-1 | Genotype-1 |
| 27 | JQ045626 | Vietnam-2011 | DENV-1 | Genotype-1 |
| 28 | FJ390386 | Vietnam-2007 | DENV-1 | Genotype-1 |
| 29 | AF180817 | Thailand-1964 | DENV-1 | Genotype-2 |
| 30 | EU863650 | Chile-2002 | DENV-1 | Genotype-4 |
| 31 | JQ915072 | South Pacific-2007 | DENV-1 | Genotype-4 |
| 32 | DQ672562 | USA:Hawaii-2001 | DENV-1 | Genotype-4 |
| 33 | AB204803 | Japan-2004 | DENV-1 | Genotype-4 |
| 34 | JN544411 | Singapore-2011 | DENV-1 | Genotype-4 |
| 35 | DQ672564 | USA:Hawaii-2001 | DENV-1 | Genotype-4 |
| 36 | U88535 | Western Pacific-1997 | DENV-1 | Genotype-4 |
| 37 | KP723473 | China:Guangzhou-2014 | DENV-1 | Genotype-5 |
| 38 | FJ390379 | USA-1998 | DENV-1 | Genotype-5 |
| 39 | GQ357692 | Singapore-2008 | DENV-1 | Genotype-5 |
| 40 | KF289072 | India-2011 | DENV-1 | Genotype-5 |
| 41 | KT827374 | China:Guangzhou-2014 | DENV-1 | Genotype-5 |
