# Supplementary material for: Molecular Characterization and Phylogenetic Analysis of the 2019 Dengue Outbreak in Wenzhou, China
Source: Front Cell Infect Microbiol. 2022 May 19;12:829380. doi: 10.3389/fcimb.2022.829380 (PMC9161089; doi:10.3389/fcimb.2022.829380)
Supplement: Supplementary Table 3 — P-value of recombination analysis. [file Table_3.docx]

**TABLE S3.** P-value of recombination analysis.

| EVENT  No. | Recombinant  sequence | Break point position | Parental sequence | Detection methods | | | | | | |
| --- | --- | --- | --- | --- | --- | --- | --- | --- | --- | --- |
|  |  | Begin/End | Major/Minor | RDP | GENECONV | BootScan | Maxchi | Chimaera | SiScan | 3Seq |
| 1 | Cam-11 | 3994/5583 | U88535/ZJWZ-62 | 1.45E  -15 | 4.47E  -23 | 3.67E  -13 | 8.45E  -15 | 2.83E  -14 | 2.30E  -20 | 1.47E  -12 |
| 2 | Cam-11 | 408/1070 | U88535/ZJWZ-18 | 2.96E  -24 | 1.60E  -18 | 2.86E  -16 | 3.20E  -11 | 2.03E  -06 | 7.05E  -11 | 1.47E  -12 |
| 3 | Cam-11 | 2549/3165 | U88535/ZJWZ-18 | 3.20E  -19 | 5.04E  -17 | 9.25E  -16 | 3.11E  -11 | 1.11E  -10 | 2.25E  -10 | 1.47E  -12 |
| 4 | Cam-03 | 9540/10066 | U88535/ZJWZ-18 | 9.59E  -15 | 1.36E  -13 | 3.09E  -14 | 2.64E  -05 | 1.06E  -10 | 1.69E  -08 | 2.37E  -10 |
| 5 | Cam-03 | 5670/6106 | Cam-11/ZJWZ-18 | 3.08E  -14 | 2.21E  -11 | 3.08E  -14 | 1.62E  -05 | 1.38E  -04 | 8.49E  -03 | 2.79E  -09 |
| 6 | Cam-03 | 8001/8536 | U88535/ZJWZ-18 | 5.17E  -12 | 1.85E  -08 | 2.03E  -09 | 4.14E  -07 | 7.32E  -05 | 6.74E  -04 | 3.92E  -10 |
| 7 | Cam-03 | 202/407 | Cam-11/JQ045626 | 8.36E  -14 | 1.58E  -12 | - | 4.31E  -04 | 4.26E  -04 | - | 3.67E  -08 |
| 8 | Cam-03 | 6756/7096 | U88535/ZJWZ-18 | 1.16E  -11 | 1.65E  -08 | 5.50E  -10 | 4.37E  -04 | 9.63E  -03 | - | 7.31E  -07 |
| 9 | Cam-11 | 1550/1804 | U88535/ZJWZ-18 | 5.55E  -08 | - | 1.11E  -03 | - | - | - | 6.27E  -04 |
| 10 | KJ755855 | 7664/7924 | DQ193572/Unknow | 8.68E  -04 | - | - | 3.45E  -02 | - | 2.75E  -02 | - |
