# Supplementary material for: Molecular Characterization and Phylogenetic Analysis of the 2019 Dengue Outbreak in Wenzhou, China
Source: Front Cell Infect Microbiol. 2022 May 19;12:829380. doi: 10.3389/fcimb.2022.829380 (PMC9161089; doi:10.3389/fcimb.2022.829380)
Supplement: Supplementary Table 4 — Padj-value of recombination analysis. [file Table_4.docx]

**TABLE S4.** Padj-value of recombination analysis.

| EVENT  No. | Recombinant  sequence | Break point position | Parental sequence | Detection methods | | | | | | |
| --- | --- | --- | --- | --- | --- | --- | --- | --- | --- | --- |
|  |  | Begin/End | Major/Minor | RDP | GENECONV | BootScan | Maxchi | Chimaera | SiScan | 3Seq |
| 1 | Cam-11 | 3994/5583 | U88535/ZJWZ-62 | 4.83E  -15 | 3.58E  -22 | 5.87E  -13 | 7.61E  -14 | 2.26E  -13 | 1.61E  -19 | 4.41E  -12 |
| 2 | Cam-11 | 408/1070 | U88535/ZJWZ-18 | 2.96E  -23 | 6.40E  -18 | 2.29E  -15 | 9.60E  -11 | 4.06E  -06 | 2.47E  -10 | 4.41E  -12 |
| 3 | Cam-11 | 2549/3165 | U88535/ZJWZ-18 | 1.60E  -18 | 1.34E  -16 | 3.70E  -15 | 9.60E  -11 | 2.96E  -10 | 5.25E  -10 | 4.41E  -12 |
| 4 | Cam-03 | 9540/10066 | U88535/ZJWZ-18 | 2.40E  -14 | 2.72E  -13 | 6.18E  -14 | 3.96E  -05 | 2.96E  -10 | 2.96E  -08 | 5.33E  -10 |
| 5 | Cam-03 | 5670/6106 | Cam-11/ZJWZ-18 | 6.16E-14 | 2.95E  -11 | 6.18E  -14 | 2.92E  -05 | 1.84E  -04 | 9.91E  -03 | 4.19E  -09 |
| 6 | Cam-03 | 8001/8536 | U88535/ZJWZ-18 | 7.39E  -12 | 1.85E  -08 | 2.32E  -09 | 9.32E  -07 | 1.17E  -04 | 9.44E  -04 | 7.06E  -10 |
| 7 | Cam-03 | 202/407 | Cam-11/JQ045626 | 1.39E  -13 | 2.53E  -12 | - | 4.92E  -04 | 4.87E  -04 | - | 4.72E  -08 |
| 8 | Cam-03 | 6756/7096 | U88535/ZJWZ-18 | 1.45E  -11 | 1.85E  -08 | 7.33E  -10 | 4.92E  -04 | 9.63E  -03 | - | 8.22E  -07 |
| 9 | Cam-11 | 1550/1804 | U88535/ZJWZ-18 | 6.17E  -08 | - | 1.11E  -03 | - | - | - | 6.27E  -04 |
| 10 | KJ755855 | 7664/7924 | DQ193572/Unknow | 8.68E  -04 | - | - | 3.45E  -02 | - | 2.75E  -02 | - |
